# Pathways from maternal depressive symptoms to children’s academic performance in adolescence: A 13‐year prospective‐longitudinal study

**DOI:** 10.1111/cdev.13685

**Published:** 2021-10-22

**Authors:** Laura Bechtiger, Annekatrin Steinhoff, Jessica M. Dollar, Simone E. Halliday, Susan P. Keane, Susan D. Calkins, Lilly Shanahan

**Affiliations:** ^1^ Jacobs Center for Productive Youth Development University of Zurich Zurich Switzerland; ^2^ Department of Human Development and Family Studies University of North Carolina at Greensboro Greensboro North Carolina USA; ^3^ Department of Educational Science University of Bern Bern Switzerland; ^4^ Department of Psychology University of North Carolina at Greensboro Greensboro North Carolina USA; ^5^ Department of Psychology University of Zurich Zurich Switzerland

## Abstract

The pathways through which exposure to maternal depressive symptoms in early childhood are linked to academic performance during adolescence are poorly understood. This study tested pathways from maternal depressive symptoms (age 2–5) to adolescent academic performance (age 15) through cumulative parenting risk (age 7) and subsequent child functioning (age 10), using multi‐informant data from a prospective longitudinal community study spanning 13 years (*N* = 389, 47% male, 68% White). Structural equation models testing indirect effects revealed small associations between maternal depressive symptoms and increased cumulative parenting risk and poorer child functioning, and, via these pathways, with poorer academic performance. Thus, childhood exposure to maternal depressive symptoms may be associated with pathways of risk that could limit children's educational opportunities.

**Abbreviations**
CBCL: Child Behavior Checklist
CFI: comparative fit index
CHAOS: Confusion, Hubbub, and Order Scale
D‐KEFS: Delis‐Kaplan Executive Function System
FIML: full information maximum likelihood
GPA: grade point average
RMSEA: root mean square error approximation
SCL‐90R: Symptom Checklist‐90 Revised
SES: socioeconomic status
SRMR: square root mean residual
WIAT‐II: second edition of the Wechsler Individual Achievement Test

During their reproductive years, women are at increased risk of experiencing elevated symptoms of depression (Kessler, 2006), including negative affect, lack of motivation or joy, and difficulties with emotion regulation. Though the etiology of depression in women is complex, stressful family life events, the mental and physical labor of child care, and potential role conflicts (being an employee, a partner, a mother) likely contribute to decreases in mothers’ mental well‐being (Ciciolla & Luthar, 2019; Nomaguchi & Milkie, 2020). Accordingly, as many as one in five children in the United States are exposed to their mothers’ clinically relevant symptoms of depression, and many more experience maternal subclinical depressive symptoms (Campbell et al., 2009). Maternal depressive symptoms have been linked to poorer well‐being and social functioning in their children—including emotional and behavioral problems (e.g., Campbell et al., 2009) and poorer academic performance (Mensah & Kiernan, 2010). Cross‐sectional and short‐term longitudinal research focusing on the elementary school years shows that higher levels of maternal depressive symptoms are associated with less school readiness (Baker & Iruka, 2013), poorer cognitive development such as fewer verbal abilities (Ahun et al., 2017), and poorer academic performance (Claessens et al., 2015; Mensah & Kiernan, 2010) in their children.

Emerging evidence suggests that exposure to maternal depressive symptoms in childhood may also have long‐lasting negative associations with cognitive functioning (Wu et al., 2018), and adolescent academic performance (Pearson et al., 2016; Shen et al., 2016). Adolescent academic performance (i.e., grade point average [GPA] and results on standardized achievement tests) is crucial not only for later educational, career, and income opportunities, but also for positive adjustment in other life domains. In fact, for these outcomes, school achievement is more crucial than cognitive functioning per se, likely because school achievement captures cognitive and personality traits and also social adaptiveness and other skills that are relevant for life success (Borghans et al., 2016). Therefore, it is of special importance to understand early risk processes that contribute to academic performance inequalities in adolescence. However, research on the mechanisms by which maternal depression has long‐term effects on children's academic performance is scarce. Even though there is a genetic component to the intergenerational transmission of (depression) risk, evidence suggests that maternal depressive symptoms also convey an environmental risk to children's adjustment across domains, likely through family and parenting processes (Hails et al., 2019; Natsuaki et al., 2014).

In this study, we examine whether the association of exposure to maternal depressive symptoms in the preschool period (ages 2–5) and adolescent academic performance (age 15) can be explained by a chain of events that begins with an *accumulation* of parenting behaviors and aspects of the home environment (age 7) that have been associated with children's poorer academic adjustment. These include unsupportive learning environments (i.e., non‐stimulating, chaotic surroundings), a negative emotional climate (i.e., high on hostility and stress), inconsistency, and low involvement in children's education. Together, we refer to these behaviors as cumulative parenting risk. Cumulative parenting risk may lead to subsequent poorer child adaptive school behaviors and behavioral regulation, and more mental health problems (age 10) which are associated with children's poorer academic performance. We refer to these aspects of child adjustment as child functioning.

## Long‐term associations between maternal depressive symptoms and children's academic performance

Few long‐term longitudinal studies have examined associations between children's exposure to maternal depression and their academic performance in adolescence. One study revealed an association between parental depressive disorders at any point in children's lives from the postnatal period to age 16 and children's lower school performance at age 16 (Shen et al., 2016). Other work has documented longitudinal associations between postnatal depression (i.e., in the first year after birth) in mothers and children's academic performance in adolescence (Murray et al., 2010; Pearson et al., 2016; Psychogiou et al., 2019). However, recent evidence highlights that the years preceding school entry constitute a particularly sensitive period for a potential negative influence of maternal depression on child adjustment (Wall‐Wieler et al., 2020). Early childhood (i.e., under 6 years of age) is a developmental period characterized by rapid brain development and learning during which children may be especially susceptible to stress, including experiences of family adversity (Black et al., 2017; Shonkoff & Garner, 2012).

Parents’ behaviors and cognition in early childhood are important determinants of children's academic socialization (Taylor et al., 2004). Mothers with high levels of depressive symptoms may struggle to adequately provide material and emotional support that children need during the transition to school. Indeed, maternal depressive symptoms before school entry have been associated with academic achievement in preadolescence through children's poorer cognitive and behavioral engagement with school (Ahun et al., 2020). Thus, maternal depression in the preschool period can initiate pathways to children's poorer academic performance.

The limited existing evidence on postnatal depression further indicates that parenting behaviors (i.e., negativity, less provision of cognitive support; Murray et al., 2010; Psychogiou et al., 2019) and various forms of child functioning (i.e., executive function skills, mental health; Pearson et al., 2016; Psychogiou et al., 2019) may be involved in these pathways. For example, one study found that the association between postnatal maternal depressive symptoms and children's academic performance at age 16 was at least partially explained by negativity in the parent–child relationship at age 7, which, in turn, was associated with more child mental health problems at age 10 (Psychogiou et al., 2019).

However, these studies had a narrow focus on specific parenting behaviors and did not consider that maternal depressive symptoms likely have cross‐cutting effects on a variety of parenting behaviors (see Goodman et al., 2020 for a review). Research has further shown that not all mothers with symptoms of depression show the same pattern of adverse parenting behavior (Wang & Dix, 2013). A cumulative approach to parenting risk can (1) account for the pervasiveness of maternal depressive symptoms on parenting behaviors, and (2) capture the heterogeneity of impaired parenting behavior in the context of maternal depressive symptoms.

## Pathway a: Maternal depression and cumulative parenting risk

The most direct pathway linking maternal depression to adolescent academic performance is via impaired parenting behaviors (Goodman et al., 2020). Parenting behaviors focused on creating a supportive learning environment (i.e., stimulating, non‐chaotic surroundings), a safe and positive emotional climate (i.e., low on hostility and stress), consistency, and involvement in children's education are known to predict children's academic performance (Boonk et al., 2018; Burchinal et al., 2002; Pinquart, 2016). Since mothers are typically “household captains” (Ciciolla & Luthar, 2019) who carry the mental burden of organizing the home environment and coordinating the family's routines and schedules, including their children's schooling, maternal depressive symptoms may negatively affect children's development through a lack of encouraging parenting behaviors and the presence of more negative parenting behaviors.

Indeed, mothers with high levels of depressive symptoms may have difficulty engaging in supportive parenting behaviors, establishing stimulating environments and a positive emotional climate that promote their children's transition to the school environment. Prior research has shown that maternal depression in early childhood is associated with less maternal involvement in children's education (Kohl et al., 2000), less consistency in enforcing rules and discipline (Letourneau et al., 2010), and a more hostile and stressed home environment (Lovejoy et al., 2000). More chaos in the home—including with respect to school‐related matters (Hur & Buettner, 2015)—and a less emotionally and cognitively stimulating environment (Baker & Iruka, 2013) have even been shown to partially explain the association between maternal depression and children's school readiness. For emotional and cognitive stimulation, this appears to be the case throughout childhood (Wu et al., 2018).

**FIGURE 1:**
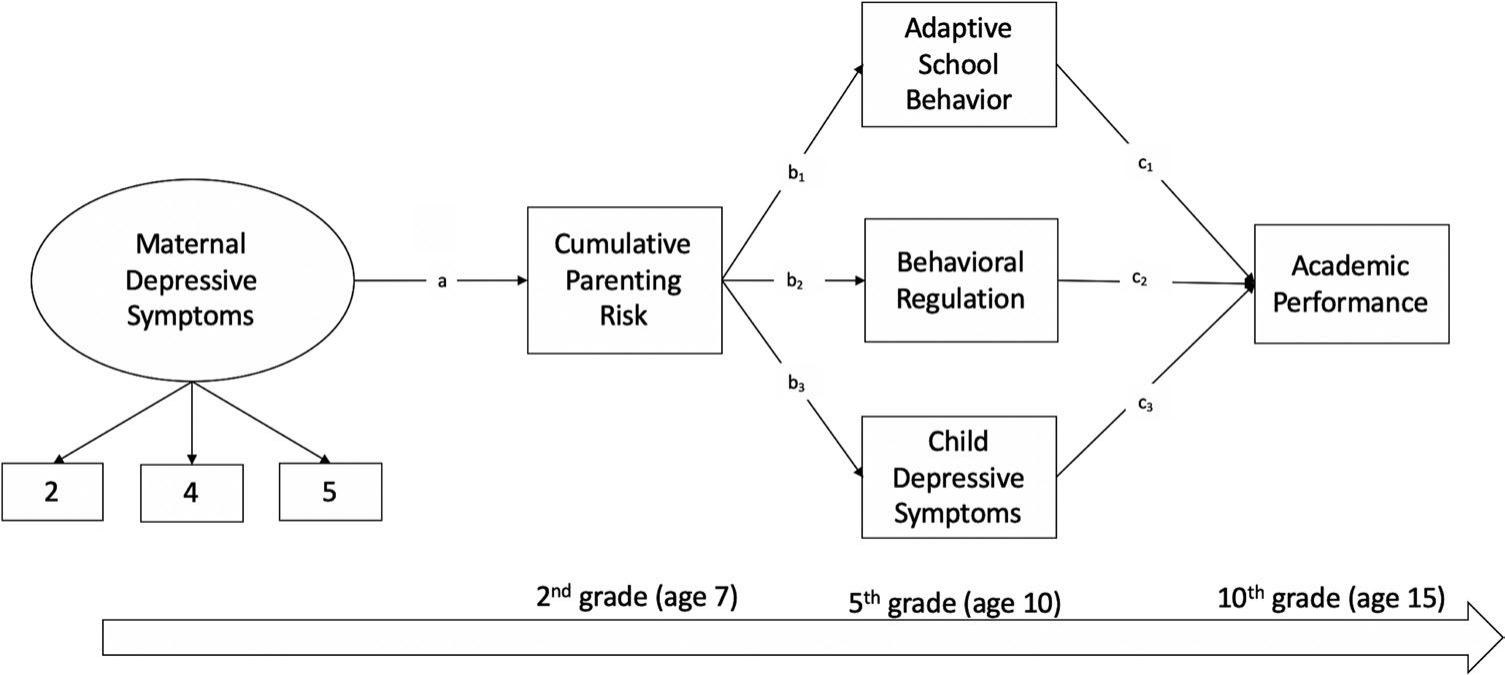
Hypothesized indirect paths from maternal depressive symptoms in childhood to adolescent academic performance

Drawing from a cumulative risk approach (Evans et al., 2013), we assume that the *accumulation* of such parenting risk behaviors in the context of elevated maternal depressive symptoms may impair the development of child functioning that is important for adolescent academic performance. We assume that the higher mothers’ depressive symptom load in the preschool period, the more parenting behaviors are impaired. Parenting risk is important for children's school adjustment around the time of school entry, when parents play an important role in helping children navigate the task of adapting to the school environment and forming long‐lasting school‐relevant habits (Taylor et al., 2004). We hypothesize a chain of events linking maternal depression to children's adolescent academic performance that begins with a link between higher levels of maternal depressive symptoms and more cumulative parenting risk (path “a” in Figure 1).

## Pathway b and c: Maternal parenting risk to children's functioning and academic performance

In turn, cumulative parenting risk in the context of maternal depressive symptoms could have long‐lasting downstream associations with children's functioning, which we define to encompass adaptive school behaviors, behavioral regulation, and mental health (paths “b_1_” to “b_3_” in Figure 1). These domains of child functioning have been linked to parenting behaviors that are associated with children's academic performance.

First, parental involvement in school and a stimulating home environment have been associated with indicators of adaptive school behaviors (El Nokali et al., 2010; Son & Morrison, 2010). Adaptive school behaviors, such as following classroom rules, interacting respectfully with peers and teachers, communicating needs clearly, learning strategies to solve new academic problems, and adapting to unexpected and new situations, are important behaviors in the school context and are positively associated with academic performance (Gajda et al., 2017; Wentzel, 1991).

Second, parenting, such as inconsistent discipline, has also been associated with children's self‐regulation problems (Roskam et al., 2014). Children's self‐regulatory skills have further been shown to mediate the association between home chaos and disengagement with school (Garrett‐Peters et al., 2019). Behavioral regulation is a component of self‐regulation that is particularly important for school success (McClelland & Cameron, 2011; Sektnan et al., 2010). The control of attention and inhibition (e.g., focusing one's attention on the classwork instead of talking with a friend during class or raising one's hand before speaking instead of interrupting the class) may be especially relevant in the school context.

Third, harsh and inconsistent parenting and a chaotic home environment, have been shown to mediate the association between maternal depression and child depressive symptoms (Evans et al., 2005; Goodman et al., 2020). Children with elevated depressive symptoms may experience anhedonia (i.e., lack of motivation and pleasure), rumination (i.e., thoughts that constantly revolve around the same issue), and somatic complaints (e.g., unexplained pains). These symptoms could place them at risk of poor academic performance (Dalsgaard et al., 2020). For example, children with these symptoms may be less engaged with school and have difficulty deriving pleasure from school activities. Depressive symptoms are likely to alter children's attention processes and lead to the adoption of maladaptive learning strategies (Moilanen et al., 2010). Children with somatic complaints may also be more likely to miss school (Roth‐Isigkeit et al., 2005), leading to further disengagement and missed coursework.

Adaptive school behavior and behavioral regulation have often been considered as indicators of school functioning in the context of the early school years. However, they may still be important in the period before the transitions to the middle and high school years, as the higher workload and stronger focus on independent learning in secondary school require higher organizational and cognitive skills. Therefore, we hypothesize that these aspects of child functioning and also depressive symptoms in the fifth grade are related to adolescent academic performance (paths “c_1_” to “c_3_” in Figure 1).

## Sex differences

Some prior studies have identified sex differences in the association between maternal depression and adolescent academic achievement. For example, some studies found that maternal depression was more strongly associated with poor school performance in female than in male children (Ahun et al., 2020; Shen et al., 2016). In contrast, other studies reported that maternal depression was associated with poorer academic performance (Murray et al., 2010) and higher school dropout (Ensminger et al., 2003) in adolescent males but not in females. The vastly different methodologies and sample characteristics of the studies (i.e., high‐risk neighborhood vs. nationally representative sample; cohort study vs. register‐based study) make it difficult to compare results. Sex differences may also emerge in some of the mediating pathways. For example, after puberty, depressive symptoms are more common in female than male adolescents (which could mediate the pathway from maternal depressive symptoms in preschool to adolescent academic performance; Hankin et al., 2015). Therefore, sex differences in the association between maternal depressive symptoms and later academic performance in adolescent children need further investigation.

## Covariates

Although maternal depression cuts across sociodemographic groups, single mothers and mothers from a lower socioeconomic background (e.g., low education, low income) are more likely to experience elevated symptoms of depression (Ertel et al., 2011; Goodman et al., 2011; Pascoe et al., 2006). These sociodemographic markers not only characterize contexts in which maternal depressive symptoms are more likely to emerge, but have also been associated with poorer academic performance in children (Battle & Lewis, 2002; Jeynes, 2005). There is inconsistent evidence on race or ethnicity and maternal depressive symptoms (Ertel et al., 2011; Horwitz et al., 2007). In addition, children's externalizing symptoms are associated with both, mothers’ elevated depressive symptoms (e.g., Shaw et al., 2016) and children's poorer academic performance (Masten et al., 2005). In other words, children's early externalizing symptoms could account for the association between maternal depressive symptoms and children's academic performance. Therefore, we adjust our analyses for socioeconomic status (SES), race, single parenthood, and early childhood externalizing symptoms to account for their potential confounding role in the association between maternal depressive symptoms and children's adolescent academic performance.

## The current study

The emerging evidence of long‐term associations between maternal depression and academic performance in adolescent children needs to be supported by studies that assess symptoms (and not only diagnoses) of maternal depression (cf. Shen et al., 2016) during the years immediately preceding school entry (rather than during the postnatal period only; Pearson et al., 2016; Psychogiou et al., 2019). We extended the scope of research on this topic by focusing on maternal depressive symptoms in the preschool period and assessing cumulative parenting risk and a wider range of child functioning.

As illustrated in Figure 1, we hypothesized that (1) exposure to elevated maternal depressive symptoms from preschool to first grade increases the risk of greater parenting risk (path “a”); (2) accumulated parenting risk during a developmental period that includes important school transitions may cause children to miss out on opportunities that support their ongoing maturation, such as the development of adaptive school behaviors, behavioral regulation, and mental health (paths “b_1_” to “b_3_”); and (3) child adaptive school behaviors, behavioral regulation, and mental health during the transition to middle school predict adolescent academic performance (paths “c_1_” to “c_3_”). Specifically, in a confirmatory analysis of the specified hypotheses, we examined a path model from elevated depressive symptoms in mothers during their children's childhood to cumulative parenting risk during the early school years to poorer child functioning in late middle childhood and lower academic performance (GPA and objective achievement test) during the adolescent years. We further conducted explorative analyses to examine potential sex differences in these pathways. Explorative post hoc analyses were conducted to examine the role of continued maternal depressive symptoms and the stability of academic performance across the school years.

## METHODS

The current study utilized data from three cohorts of children who are part of an ongoing longitudinal study of social and emotional development. The goal for recruitment was to obtain a sample of children who were at risk for developing future externalizing behavior problems, and who were representative of the surrounding community (i.e., a mid‐sized city in North Carolina) in terms of race and SES. All cohorts were recruited through child daycare centers, the County Health Department, and the local Women, Infants, and Children program. Potential participants for cohorts 1 and 2 were recruited at 2 years of age (cohort 1: 1994–1996 and cohort 2: 2000–2001) and screened using the Child Behavior Checklist (CBCL 2–3; Achenbach, 1992), completed by the mother, in order to over‐sample for externalizing behavior problems. Children were identified as being at risk for future externalizing behaviors if they received an externalizing *T*‐score of 60 or above. Efforts were made to obtain approximately equal numbers of males and females. This recruitment effort resulted in a total of 307 children. Cohort 3 was initially recruited when infants were 6 months of age (in 1998) for their level of frustration, based on laboratory observation and parent report, and were followed through the toddler period (see Calkins et al., 2002, for more information). Children from Cohort 3 whose mothers completed the CBCL at 2 years of age (*N* = 140) were then included in the larger study. Of the entire sample (*N* = 447), 37% of children were identified as being at risk for future externalizing problems. There were no significant demographic differences between cohorts with regard to gender, *χ* ^2^(2, *N* = 447) = 0.63, *p* = .73, race, *χ* ^2^(2, *N* = 447) = 1.13, *p* = .57, or 2‐year SES, *F*(2, 444) = .53, *p* = .59.

Of the 447 originally selected participants, six were dropped because they did not participate in any data collection at 2 years old. An additional 12 families participated at recruitment, did not participate at 2 years, but did participate at later years. At 4 years of age, 399 families participated. Families lost to attrition included those who could not be located, moved out of the area, declined participation, or did not respond to phone and letter requests to participate. There were no significant differences between families who did and did not participate at age four in terms of gender, *χ* ^2^(1, *N* = 447) = 3.27, *p* = .07, race, *χ* ^2^(1, *N* = 447) = 0.65, *p* = .42, 2‐year SES, *t*(432) = −0.92, *p* = .36, 2‐year externalizing *T* score, *t*(445) = 0.45, *p* = .65, or maternal depressive symptoms at age 2, *t*(24) = −0.39, *p* = .70. At age 5, 365 families participated, including four that did not participate in the 4‐year assessment. Again, there were no significant differences between families who did and did not participate in terms of gender, *χ* ^2^(1, *N* = 447) = 0.76, *p* = .38, race, *χ* ^2^(1, *N* = 447) = 0.14, *p* = .71, 2‐year SES, *t*(432) = −1.99, *p* = .05, 2‐year externalizing *T* score, *t*(445) = 1.39, *p* = .17, or maternal depressive symptoms at age 2, *t*(66) = −0.65, *p* = .52. At 7 years of age, 350 families participated, including 19 that did not participate in the 5‐year assessment. Again, there were no significant differences between families who did and did not participate in terms of gender, *χ* ^2^(1, *N* = 447) = 2.12, *p* = .15, race, *χ* ^2^(3, *N* = 447) = 1.47, *p* = .69, 2‐year externalizing *T* score, *t*(445) = 1.30, *p* = .19, or maternal depressive symptoms at age 2, *t*(92) = −0.35, *p* = .73. Families with lower 2‐year SES, *t*(432) = −2.61, *p* < .01, were less likely to participate in the 7‐year assessment. At age 10, 357 families participated, including 31 families that did not participate in the 7‐year assessment. No significant differences were noted between families who did and did not participate in the 10‐year assessment in terms of child gender, *χ* ^2^(1, *N* = 447) = 3.31, *p* = .07; race, *χ* ^2^(3, *N* = 447) = 5.78, *p* = .12; 2‐year SES, *t*(432) = 0.02, *p* = .98; 2‐year externalizing *T* score, *t*(445) = −0.11, *p* = .91, or maternal depressive symptoms, *t*(102) = −1.75, *p* = .08. At age 15, 327 families participated, including 27 families that did not participate in the 10‐year assessment. There were no significant differences between families who did and did not participate in the 15‐year assessment in terms of race *χ* ^2^(3, *N* = 447) = 3.96, *p* = .27; 2‐year SES *t*(432) = −0.56, *p* = .58; 2‐year externalizing *T* score *t*(445) = 0.24, *p* = .81, or maternal depressive symptoms at age 2, *t*(152) = −1.10, *p* = .27. Boys were less likely to participate in the 15‐year assessment *χ* ^2^(1, *N* = 447) = 9.31, *p* = .002.

The analytic sample in this study consists of 389 participants. Males and females are equally represented (47% males). Two‐thirds (67.6%) of the sample is White, 25.7% of the sample is Black, and 6.6% mixed race or of another race or ethnicity. The current sample includes families from all social strata (range of Hollingshead index: 14–66; *M* = 39.51, *SD* =11.13, with scores from 40 to 54 representative of middle class). We used data from maternal assessments at child ages 2, 4, 5, and 7 (second grade), teacher assessments at ages 7, 10 (fifth grade), and 15 (tenth grade), and child assessments at ages 10 and 15.

### Measures

#### Maternal depressive symptoms in preschool (ages 2–5)

Maternal depressive symptoms were assessed at child ages 2, 4 (Cohorts 1 and 2 only), and 5 (*α* =.868, *α* =.907, *α* =.885, respectively) using the depression subscale of the Symptom Checklist‐90 Revised (SCL‐90R), a self‐report measure that assesses a broad range of psychopathological symptoms (Derogatis, 1994). Mothers rated whether 90 symptoms had caused them distress over the previous 7 days on a 5‐point scale, ranging from 0 = not at all to 4 = extremely. The depression subscale of the SCL‐90R consists of 13 items, which assess symptoms such as lack of interest and motivation, low energy, and feelings of hopelessness (e.g., “feeling low in energy or slowed down,” “feelings of worthlessness”). We used these three assessments to model a latent factor of maternal depressive symptoms that could capture stable levels of depressive symptoms during the early childhood period.

#### Parenting risk in second grade (age 7)

A cumulative score of *parenting risk* consisting of six indicators was created. First, *low parental school involvement* was teacher‐rated using 13 items (*α* =.894) from the teacher version of the Parent Involvement Questionnaire (Webster‐Stratton et al., 2001), which assesses the extent of parents’ home‐ and school‐based involvement on a 5‐point response scale from 1 = never to 5 = more than once a week. Example items included “parent is interested in knowing the teacher,” “parent encourages education,” and “parent attended school meetings.” Items were averaged, and scores were reversed, with higher values representing less involvement.

Second, *low stimulation in home environment* was measured using the middle childhood version of the Home Observation for Measurement of the Environment (Caldwell & Bradley, 1984) scale. The total score consisted of the sum of 59 items, 40 of which were completed by the primary caregiver and 19 of which were rated by home interviewers (*α* = .752). Each item was rated using either 0 = no or 1 = yes. Example items included “family has fairly regular and predictable daily schedule for child,” “family requires child to carry out certain self‐care routines,” and “family has a TV, and it is used judiciously and not left on continuously.” The score was reversed, with higher values representing less stimulating home environment.

Third, *chaotic home environment* was assessed with the Confusion, Hubbub, and Order Scale (CHAOS; Matheny et al., 1995), a 15‐item true‐or‐false questionnaire completed by the primary caregiver (*α* = .817). Example items included “it's a real zoo in our home” and “you can't hear yourself think in our home.” Items were summed. This scale was assessed in Cohorts 2 and 3 only. A sensitivity analysis for which the parenting risk score was computed without the CHAOS measure can be found in Figure S1 (results remained stable).

Fourth, *inconsistent discipline* was assessed using the inconsistent discipline subscale of the Alabama Parenting Questionnaire (Shelton et al., 1996). Mothers were asked to rate the frequencies of six behaviors on a scale of 1–5 (1 = never, 5 = always; *α* = .714). Example items included “you threaten to punish your child and then do not actually punish him/her,” and “your child talks you out of being punished after he/she has done something wrong.” Items were averaged.

Fifth, *parenting stress* was measured with the Parenting Stress Index Short Form (Abidin, 1995). The total stress score consists of the sum of 36 items from three subscales (i.e., parental distress, parent–child dysfunctional interaction, and difficult child) rated on a scale from 1 = strongly agree to 5 = strongly disagree (*α* = .922). Example items included “I feel trapped by my responsibilities as a parent,” “my child smiles at me much less than I expected,” and “I feel that my child is very moody and easily upset.”

Sixth, a composite mean score for *observed maternal hostility* was created based on three laboratory tasks: the craft task (6 min), the craft clean‐up task (5 min), and the puzzle box task (5 min; Eisenberg et al., 1997). During the craft task, mother and child were instructed to work on a craft project together. For the craft clean‐up task, children were instructed to clean up materials used in the craft task. For the puzzle box task, mothers instructed their children on how to put together a puzzle that the children could not see because it was in a covered box (even though the puzzle could be easily revealed). Maternal hostility reflects mothers’ expressions of anger, discounting of the child, or annoyance. Mothers scoring low on this scale were supportive in a neutral or warm manner. In contrast, mothers scoring high on this scale overtly rejected their children, blamed them for mistakes, or explicitly showed a lack of emotional support for their children. Two coders were trained to independently code maternal hostility. Their reliability was calculated for 21% of the puzzle box tasks (weighted *κ* = .74) and for 17% of both the craft task (weighted *κ* = .90) and the craft clean‐up task (weighted *κ* = .79).

Based on common practice in the cumulative risk literature (Burchinal et al., 2000; Evans et al., 2013), a binary score was created for every parenting risk indicator. Those who scored in the upper 25% on each scale were considered high risk and assigned a score of 1. The sum of these binary variables was calculated and used as the indicator of cumulative parenting risk (range 0–5; mean = 1.25, median = 1). Bivariate correlations between the individual continuous parenting indicators are presented in Supplement 2 (range of *r* = −.01 to .49).

#### Child functioning in fifth grade (age 10)

*Child adaptive school behaviors* were teacher‐rated on the adaptive skills composite of the Teacher Rating Scale in the second edition of the Behavior Assessment System for Children (Reynolds & Kamphaus, 2004), which consists of five subscales: (1) functional communication (10 items; *α* = .842; e.g., “responds appropriately when asked a question”), (2) leadership skills (6 items; *α* = .870; e.g., “is good at getting people to work together”), (3) study skills (7 items; *α* = .902; e.g., “analyzes the nature of a problem before starting to solve it”), (4) social skills (8 items; *α* = .933; e.g., “says please and thank you”), and (5) adaptability (8 items; *α* = .851; e.g., “adjusts well to new teachers”). All items were rated on a four‐point scale from 0 = never to 3 = always. The adaptive skills composite used in our analyses comprises all six subscales. Normative T scores representative of the U.S. population were used for the current study.

*Behavioral regulation* was assessed in the laboratory using the color–word task of the Delis‐Kaplan Executive Function System (D‐KEFS; Delis et al., 2001). This task is based on a Stroop procedure. Participants were presented with color words in dissonant colored ink and asked to inhibit the overlearned verbal response of reading the word in order to name the color of the ink. The speed at which the participant completed the task indicated his or her behavioral regulation ability. Scores were derived based on the normative data in the D‐KEFS scoring manual. Higher scores reflected better behavioral regulation skills.

*Child depressive symptoms* were child‐reported using the Children's Depression Inventory (Kovacs, 1992), which contains 27 items. For each item, the child was asked to choose between three sentences to best describe his or her feelings in the past 2 weeks. The items assessed negative mood (e.g., “I am sad once in a while,” “I am sad much of the time,” “I am sad all the time”), interpersonal problems (e.g., “I like being with people,” “I do not like being with people many times,” “I do not want to be with people at all”), ineffectiveness (e.g., “I can never be as good as other kids,” “I can be as good as other kids if I want to,” “I am just as good as other kids”), anhedonia (e.g., “I have fun in many things,” “I have fun in some things,” “Nothing is fun at all”), and low self‐esteem (e.g., “nothing will ever work out for me,” “I am not sure if things will work out for me,” “things will work out for me OK”). The total score was used and items were averaged. Internal consistency was at *α* = .894.

#### Academic performance in tenth grade (age 15)

*Weighted GPA* was calculated based on recommendations from the state of North Carolina (State Board of Education & Department of Public Instruction, 2005). Specifically, weighted grades for English, math, and science were created from raw grades as follows: An additional grade point was awarded to the individual subject grades for advanced, honors, and academically gifted courses, and an additional two grade points were awarded for advanced placement and international baccalaureate courses. These weighted grades were then used to calculate the GPA.

To assess students’ *standardized achievement*, we used the second edition of the Wechsler Individual Achievement Test (WIAT‐II; Wechsler, 2005). The test was administered in the laboratory. The WIAT‐II generates two standardized composite scores: one for reading (i.e., word reading, reading comprehension, and pseudoword decoding), and one for mathematics (i.e., numerical operations and mathematical reasoning). The mean of the two scores was calculated to assess overall achievement.

#### Covariates at age 2 and post hoc variables

*Family SES* was measured using the Hollingshead index (Hollingshead, 1975), a composite four‐factor index score considering education, occupation, and sex. If two parents were present in the household, an index was calculated for each of them and these were then averaged. *Single motherhood* was operationalized as 0 = (re‐)married versus 1 = single, divorced, or separated (19%). *Child race or ethnicity* was operationalized as 0 = White (67%) versus 1 = Other (33%). *Child sex* was coded as 0 = male versus 1 = female. *Child externalizing behavior* was mother‐reported using the T score of the externalizing composite of the CBCL for 2‐ to 3‐year‐olds (Achenbach, 1992). Mothers rated 26 items (11 items from the destructive behavior subscale, *α* = .752, and 15 items from the aggressive behavior subscale, *α* = .884) related to aggressive, destructive, and oppositional behaviors on a three‐point scale from 0 = not true to 2 = often true. For the *post hoc analyses*, continued maternal depressive symptoms in second, fifth, and tenth grade were also measured on the SCL‐90‐R. Academic performance in second grade was assessed in the laboratory on the WIAT‐I (Wechsler, 1992).

### Analytic strategy

Path analyses were conducted in Mplus (Version 8). All other analyses were conducted in R (Version 3.6.2). The estimated model included paths from a latent maternal depressive symptoms factor in childhood to cumulative parenting risk in second grade; from cumulative parenting risk to child adaptive school behaviors, behavioral regulation and depressive symptoms in fifth grade; and from child functioning to weighted GPA and standardized achievement in tenth grade. It also included direct paths from maternal depressive symptoms to all indicators of child functioning, GPA, and standardized achievement, and from cumulative parenting risk to GPA and standardized achievement. Constructs measured at the same time point were allowed to covary. Covariates were added to the model in a two‐step process. First, all covariates were added as predictors of all key variables. Second, non‐significant paths (*p* > .10) from covariates to key variables were removed from the final model. Some variables (i.e., child adaptive school behaviors, behavioral regulation, child depressive symptoms, GPA, standardized achievement, SES) were rescaled to a scale from 0 to 1 to facilitate model estimation (Little, 2013).

We used maximum likelihood estimation and bootstrapped standard errors (5000 draws) for model estimation. Full information maximum likelihood (FIML) was used to handle missing data because FIML includes all available data points for model estimation (Enders & Bandalos, 2001). A non‐significant *χ* ^2^ value, comparative fit index (CFI) ≥.95, root mean square error approximation (RMSEA) ≤.06, and square root mean residual (SRMR) ≤.08 were considered an acceptable model fit (Hu & Bentler, 1999).

Potential indirect effects via all possible indirect effects were examined. To test the significance of indirect effects, we applied the product‐of‐coefficients method and report bias‐corrected bootstrapped confidence intervals (MacKinnon et al., 2004).

To examine sex differences, we estimated two multi‐group models. In the first, all paths were allowed to vary freely between males and females. In the second, all direct paths between the core study variables were constrained to be equal in males and females. The two models were then compared using several fit indices. Specifically, ΔCFI ≤ .01, ΔRMSEA ≤ .015, and ΔSRMR ≤ .01 were considered to indicate model invariance (Chen, 2007; Little, 2013).

With the described path modeling framework, we chose a confirmatory approach to model testing. The examination of sex differences and all other follow‐up analyses should be considered exploratory, as we did not specify any hypotheses.

## RESULTS

Table 1 shows the descriptive statistics for and correlations among all study variables. Significant correlations revealed small to moderate associations of maternal depressive symptoms and all hypothesized mediators and with tenth‐grade GPA and standardized achievement in the hypothesized direction.

**TABLE 1:** Means, standard deviations, and correlations of study variables

Variable	*M*	*SD*	1	2	3	4	5	6	7	8	9	10	11
1. Maternal depressive symptoms (0–4)	0.80	0.22											
2. Cumulative parenting risk (0–5)	1.25	1.27	.33***										
3. Adaptive school behaviors (0–1)	0.59	0.22	−.24***	−.32***									
4. Behavioral regulation (0–1)	0.67	0.15	−.15*	−.16**	.34***								
5. Child depressive symptoms (0–1)	0.13	0.14	.19**	.23***	−.25***	−.20***							
6. Weighted GPA (0–1)	0.59	0.22	−.14*	−.32***	.52***	.31***	−.33***						
7. Standardized achievement (0–1)	0.65	0.17	−.16*	−.41***	.50***	.47***	−.42***	.67***					
8. SES age 2 (0–1)	0.49	0.21	−.19**	−.25***	.19**	.17**	−.18***	.26***	.34***				
9. Race/ethnicity (1 = other)	0.32	0.47	.05	.18***	−.19**	−.14*	.06	−.26***	−.34***	−.21***			
10. Sex (1 = female)	0.52	0.50	−.04	−.01	.15*	−.04	.06	.11*	−.03	−.09^+^	.02		
11. Externalizing behavior age 2 (0–1)	0.36	0.15	.44***	.26***	−.27***	−.14*	.20***	−.20***	−.30***	−.17***	−.01	−.08	
12. Single parenthood (1 = single)	0.19	0.39	.21**	.25***	−.21***	−.10	.10	−.27***	−.22**	−.35***	.38***	.09	.11*

Note

Race or ethnicity: 0 = White, 1 = Other. Sex: 0 = male, 1 = female. Range of variable scale in brackets after variable names.

Abbreviations: GPA, grade point average; SES, socioeconomic status.

+ *p* < .10.

* *p* < .05.

** *p* < .01.

*** *p* < .001.

In Table 2, correlations are presented by child sex. In both males and females, higher levels of maternal depressive symptoms were significantly associated with more cumulative parenting risk and lower child adaptive school behaviors but not with lower behavioral regulation. Maternal depressive symptoms were associated with higher levels of child depressive symptoms in females but not in males. Maternal depressive symptoms were marginally associated with poorer academic performance in females but not in males. This is likely due to the smaller sample sizes as all coefficients are in the same direction as in the full sample.

**TABLE 2:** Correlations by sex

Variable	1	2	3	4	5	6	7	8	9	10	11
1. Maternal depressive symptoms (0–4)	—	.27**	−.25**	−.12	.21*	−.15^+^	−.16^+^	−.19*	−.02	.41***	.22*
2. Cumulative parenting risk (0–5)	.37***	—	−.34***	−.13	.25**	−.31***	−.38***	−.20**	.23**	.14	.26***
3. Adaptive school behavior (0–1)	−.23*	−.30**	—	.46**	−.30**	.48***	.47***	.25**	−.24**	−.20*	−.34***
4. Behavioral regulation (0–1)	−.18^+^	−.19*	.19	—	−.29***	.31***	.44***	.09	−.11	−.19*	−.10
5. Child depressive symptoms (0–1)	.14	.21*	−.23*	−.05	—	−.41***	−.37***	−.10	.10	.15+	.05
6. Weighted GPA (0–1)	−.13	−.34***	.54***	.33**	−.24*	—	.68***	.32***	−.29***	−.12	−.29***
7. Standardized achievement (0–1)	−.14	−.46***	.58***	.51***	−.48**, ***	.69***	—	.29**	−.31***	−.25**	−.20*
8. SES age 2 (0–1)	−.20*	−.31***	.15	.27**	−.30**	.21*	.39***	—	−.17*	−.11	−.34***
9. Race/ethnicity (1 = other)	.13	.13	−.14	−.19*	.02	−.22**	−.38***	−.25**, ***	—	−.02	.42***
10. Externalizing behavior age 2 (0–1)	.48***	.37***	−.32**	−.11	.31**	−.28**	−.38***	−.25***	.00	—	.14*
11. Single parenthood	.20	.26**	−.10	−.07	.19*	−.27**	−.23*	−.36***	.34***	.10	—

Note

Correlations for males are presented under the diagonal in blue, correlations for females above the diagonal in red. Race or ethnicity: 0 = White, 1 = Other. Range of variable scale in brackets after variable names.

Abbreviations: GPA, grade point average; SES, socioeconomic status.

+ *p* < .10.

* *p* < .05.

** *p* < .01.

*** *p* < .001.

**FIGURE 2:**
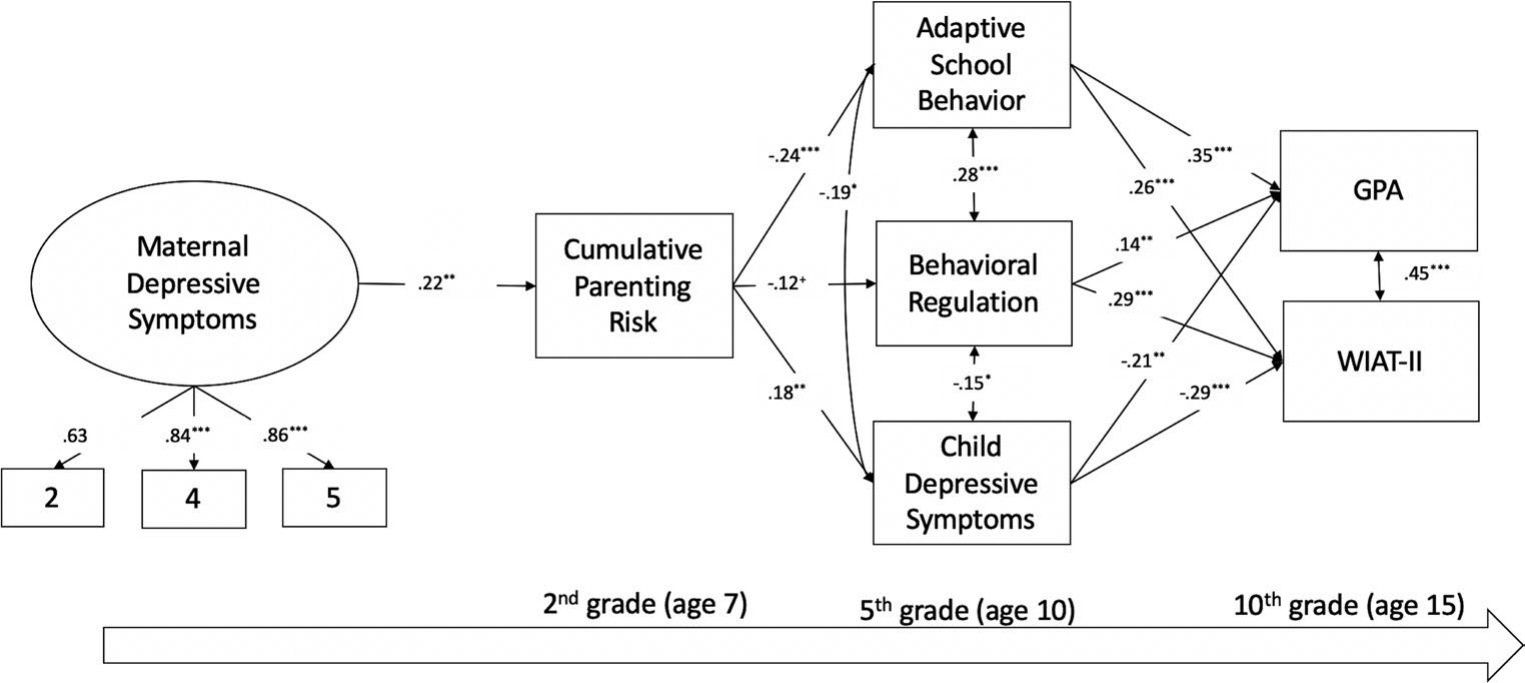
Significant standardized coefficients of the hypothesized path model. *Note*: *N* = 389; *χ* ^2^(*df*) = 60.347 (44), *p* = .0512, comparative fit index = .979, root mean square error approximation = .031, square root mean residual = .046. Direct paths from maternal depressive symptoms to child functioning and academic performance outcomes, and from cumulative parenting risk to academic performance are not shown in the figure to reduce complexity. Potential indirect effects via these direct paths were also examined. The analysis is adjusted for socioeconomic status, single motherhood, race or ethnicity (White vs. Other), sex, and child externalizing behaviors, all at age 2. ^+^ *p* < .10; *p < .05; **p < .01; ***p < .001. GPA, grade point average; WIAT‐II, second edition of the Wechsler Individual Achievement Test

Figure 2 shows the standardized coefficients from the path model. Elevated maternal depressive symptoms in childhood were significantly associated with higher levels of cumulative parenting risk at age 7. More cumulative parenting risk, in turn, was associated with lower levels of adaptive school behaviors and more depressive symptoms at age 10 and marginally associated with lower levels of behavioral regulation at that age. Higher levels of adaptive school behaviors and behavioral regulation were associated with higher GPA and standardized achievement scores, whereas higher levels of child depressive symptoms were associated with lower scores for both academic performance measures. Maternal depressive symptoms were no longer directly associated with adolescent academic achievement when covariates and indirect pathways were included in the model (see Table S3 for all standardized model coefficients, including retained covariates). Maternal depression was marginally associated with lower levels of behavioral regulation. In addition to what is shown in Figure 2, higher levels of parenting risk were independently associated with lower standardized achievement.

The total indirect effects of maternal depressive symptoms on both GPA, −.024, 95% CI (−.043, −.008), and standardized achievement, −.025, 95% CI (−.042, −.011), were significant. Table 3 shows the unique significant indirect paths that emerged from the model. We found significant indirect paths from elevated maternal depressive symptoms to (1) higher parenting risk to lower levels of adaptive school behaviors to academic performance (both GPA and standardized achievement); (2) higher parenting risk to standardized achievement; and (3) higher parenting risk to higher levels of child depressive symptoms.

**TABLE 3:** Unstandardized estimates of significant indirect effects, standard errors, and 95% bias‐corrected bootstrap confidence intervals (5000 draws)

Indirect Paths	Estimate	*SE*	Confidence intervals	
			Lower	Upper
MD → PR (2nd grade) → ASB (5th grade) → GPA (10th grade)	−.004	.002	−.008	−.001
MD → PR (2nd grade) → ASB (5th grade) → WT (10th grade)	−.002	.001	−.005	−.001
MD → PR (2nd grade) → ASB (5th grade)	−.011	.004	−.021	−.004
MD → PR (2nd grade) → CDI (5th grade)	.005	.003	.002	.012
MD → PR (2nd grade) → WT	−.006	.002	−.012	−.002

Abbreviations: ASB, adaptive school behaviors; CDI, child depression; GPA, grade point average; MD, maternal depressive symptoms; PR, cumulative parenting risk; WT, Wechsler Achievement Test Results.

We performed multi‐group analyses to test for possible sex differences in associations. Table 4 presents the model comparison between a freely estimated multi‐group model and a multi‐group model where main paths were constrained to be equal for males and females. These models did not differ significantly, suggesting no significant sex differences in the pathways.

**TABLE 4:** Model fits and comparison of multi‐group sex differences

Models	Model fit				Model difference test			
	*χ* ^2^(*df*)	CFI	RMSEA	SRMR	Δ*χ* ^2^(Δ*df*)	ΔCFI	ΔRMSEA	ΔSRMR
Free	103.953 (79)	.969	.040	.055				
Constrained	120.765 (96)	.969	.036	.062	16.812 (17)	.000	−.004	.007

Abbreviations: CFI, comparative fit index; RMSEA, root mean square error approximation; SRMR, square root mean residual.

### Post hoc analysis 1: Continuity of maternal depressive symptoms

To examine whether the pathways from maternal depressive symptoms in preschool to adolescent academic performance through cumulative parenting risk and child functioning were independent of the continuity of maternal depressive symptoms from childhood to adolescence, maternal depressive symptoms after the preschool period (i.e., in second, fifth, and tenth grade) were added to the model (see Supplement 4 for details). The main model results changed only minimally (see Table S4.1). All indirect effects from maternal depressive symptoms to adolescent academic performance in the main model remained significant (see Table S4.2). No additional indirect effects from maternal depressive symptoms in preschool to adolescent academic performance through continued maternal depressive symptoms emerged.

### Post hoc analysis 2: Continuity of academic performance

To examine whether the pathways from maternal depressive symptoms in preschool to adolescent academic performance through cumulative parenting risk and child functioning were independent from early academic performance, we included standardized achievement in second grade in the model (see Supplement 5 for details). This step attenuated some model coefficients (see Table S5.1). However, the total indirect effects from maternal depressive symptoms in preschool to adolescent academic performance and the unique indirect effect from maternal depressive symptoms through cumulative parenting risk to subsequent adaptive school behaviors to GPA remained significant (see Table S5.2). Maternal depressive symptoms in the preschool period were not significantly associated with second‐grade standardized achievement.

## DISCUSSION

Our prospective longitudinal, multi‐informant study is among the first to examine whether exposure to elevated maternal depressive symptoms during childhood is associated with children's academic high school performance approximately one decade later, in high school, and to identify developmental pathways connecting these links. Our results reveal a small bivariate association between exposure to maternal depressive symptoms during preschool and first grade and adolescent academic performance, thereby replicating prior results that link parental diagnoses of depression (Shen et al., 2016) and postnatal depression (Murray et al., 2010; Pearson et al., 2016; Psychogiou et al., 2019) to adolescent academic performance.

Our results also show that maternal depressive symptoms have the potential to initiate pathways of risk that could impair long‐term academic success. The association between maternal depressive symptoms and academic performance is explained by indirect effects via more cumulative parenting risk (e.g., chaos, hostility, low school involvement) in second grade and, in turn, lower levels of child functioning in fifth grade, which were associated with poorer academic performance in tenth grade. This extends the scope of prior research, which mostly focused on single aspects of parenting risk in the context of maternal depression, such as emotional and material support or home chaos (Hur & Buettner, 2015; Wu et al., 2018). Our results are consistent with the observation that depressive symptoms in the preschool period may reduce mothers’ capacity to bear the mental burden of supporting their children in mastering developmental tasks, such as adapting to the school context by providing a sensitive, caring, and stimulating home environment, being involved with their children's schooling, and setting consistent rules.

Indeed, the accumulation of parenting risk in second grade following elevated maternal depressive symptoms was associated with three markers of child functioning: adaptive school behaviors, behavioral regulation, and depressive symptoms. Elevated depressive symptoms may impede with mothers’ efforts to facilitate the development of child functioning in fifth grade through an accumulation of parenting risk behavior. Importantly, our results show that the *accumulation* of parenting risk is associated with poorer child functioning. In other words, mothers do not have to excel at every single parenting behavior to promote their children's academic development. Children's high levels of adaptive school behaviors and behavioral regulation and low levels of depressive symptoms likely enable children to master the educational transitions of adolescence by helping them to adapt to the rules of different teachers, focus on relevant coursework, and remain engaged with school despite the new demands and stressors of secondary school.

In addition, we identified several unique indirect effects. First, we found a significant indirect path from cumulative parenting risk following maternal depressive symptoms to children's academic performance through adaptive school behaviors in children. When parents are unable to be involved in their children's education or to provide a stimulating home environment and allow the home to become physically chaotic, children may not adequately learn the importance of focusing on and organizing their school work or following teachers’ rules. This observation is consistent with social learning theory, which emphasizes the role of behavioral reinforcement and observational learning in child development (Bandura, 1977; Patterson, 1975), and with prior evidence of a prospective association between supportive and involved parenting and child competence (Brody et al., 2002). It is also in line with evidence showing that maternal depression in the preschool period is more strongly related to children's social development than to their cognitive development (Wall‐Wieler et al., 2020).

Second, we found a significant indirect path from maternal depressive symptoms to higher parenting risk to child depressive symptoms. This is consistent with a large body of literature on the importance of parenting in the intergenerational transmission of depression from mother to child (Goodman et al., 2020). Prior research on parenting and maternal depression has often focused on emotion‐focused parenting styles, such as sensitivity or harshness. In our study, we specifically focus on the caregiving environment necessary to promote academic success. The higher levels of child depressive symptoms observed here may be attributable to children's increased difficulties adapting to the school environment, as was confirmed in our post hoc analysis: poorer academic performance in second grade was associated with more depressive symptoms in fifth grade. This corroborates prior research showing that depressive symptoms mediate the association between childhood academic success and adolescent academic success (Psychogiou et al., 2019).

Third, we also found an indirect path from maternal depressive symptoms to adolescent standardized achievement, but not GPA, via cumulative parenting risk, independent of any of the hypothesized mediating roles of child functioning. Standardized achievement is a more objective measure of academic performance compared to grades, as it is more reflective of students’ cognitive skills, such as executive function and IQ, rather than students’ behavior (Borghans et al., 2016). These “hard” cognitive skills may not have been completely captured by the aspects of child functioning included in this study, which could explain the emergence of the indirect path from maternal depressive symptoms to standardized achievement through cumulative parenting risk, independent of child functioning.

The indirect associations of maternal depressive symptoms and adolescent academic performance could not be explained by the continuity of maternal depressive symptoms across childhood nor the stability of academic performance, as shown in two post hoc analyses. First, no additional indirect paths from maternal depressive symptoms in preschool to adolescent academic performance through the continuity of maternal depressive symptoms were identified. This is consistent with prior research showing that maternal depressive symptoms may have especially detrimental consequences on child adjustment to school in the preschool period (Wall‐Wieler et al., 2020) when parental socialization is critical (Taylor et al., 2004). These findings further highlight the importance of the family and home environment early in development for child adjustment. Second, no additional pathway from maternal depressive symptoms in preschool to adolescent academic performance through early academic performance was identified. This contradicts prior research that found that maternal depression is associated with early academic performance (e.g., Mensah & Kiernan, 2010). Significant indirect paths through cumulative parenting risk and child functioning (especially adaptive school behaviors) from maternal depressive symptoms to adolescent academic performance independent of early academic performance suggest (1) that family risk may impair the development of children's soft skills, and (2) that in middle childhood, these skills are important for adolescent academic success independent of earlier academic performance.

We found no sex differences in the described processes. This compounds the mixed findings of existing studies (Ensminger et al., 2003; Shen et al., 2016). However, most previous studies focused on the main effects of maternal depression on academic performance and did not examine the pathways that might explain this link. The lack of sex differences in these pathways including parenting risk and child functioning could indicate that males and females equally rely on maternal support when adapting to the new school environment. Once in school, students are faced with similar challenges, such as having to organize their schedule or complete homework on time. Furthermore, previous studies did not adjust for childhood externalizing problems. Males are more prone to externalizing problems, which, in turn, are typically associated with poorer academic performance (Masten et al., 2005). Thus, the failure to account for childhood externalizing behaviors could have resulted in sex differences in previous work, which are accounted for by the inclusion of externalizing behavior in the current study.

Our associations are small in size. Prior studies have found associations between parental depression and adolescent academic performance that are similar in size to the association of SES and academic performance—which has been robustly established (Sirin, 2005). However, these prior studies focused solely on diagnoses of maternal depression (Shen et al., 2016). The current symptom‐level study reveals smaller long‐term associations, which could be attributed to the lower symptom burden in community mothers. Nevertheless, at every assessment from age 2 to 5, a good 10% of mothers in our sample show clinically relevant symptoms of depression. Our small effect sizes are also consistent with the literature on parenting as a mediator of the association between maternal depression and child functioning, which typically finds small effects (Goodman et al., 2020). The small effects also suggest that mothers’ depressive symptoms do not inevitably contribute to children's poorer academic development. This is consistent with the idea that not all variation in parenting behaviors is associated with observed differences in child functioning (Scarr, 1992).

### Implications

Our findings have important implications for prevention and intervention efforts. Ideally, maternal depressive symptoms would be prevented. Although the causes of maternal depression are complex, it is well‐known that mothers are often overburdened with many simultaneous roles that tend to compete for their time, including being a parent of multiple children, a partner, an employee, a household captain, a community and family liaison, a chauffeur, a family nurse and vet, and more, which could induce stress and depression in the first place (Ciciolla & Luthar, 2019; Nomaguchi & Milkie, 2020). Role overload in mothers became particularly apparent during the COVID‐19 pandemic, when it was mostly mothers who took on the additional roles of being a home teacher, coordinating with schools, and implementing new household rules; indeed, rates of maternal depression and anxiety increased during this time (American Psychological Association, 2020). Maternal overload and burnout could, in part, be prevented by increased gender equity and active involvement of partners, workplaces, and society in sharing the mental load associated with raising children and coordinating families (Nomaguchi & Milkie, 2020).

If maternal depressive symptoms are not prevented, then treating it might interrupt the chain of risk documented by our analyses. This is in line with intervention research showing that treating maternal depression improves aspects of children's functioning, such as mental health and perceived academic and social competence (Cuijpers et al., 2014; Garber et al., 2011), and that this is likely due to changes in parenting behavior (Garber et al., 2011; Goodman & Garber, 2017). Our results suggest that parenting interventions to prevent child maladjustment should target a variety of parenting behaviors such as providing parents with tools to create an organized and stimulating environment, and promoting the importance of consistency and warmth. Since children who experience an accumulation of parenting risk are at particular risk, preventing such an accumulation may be especially important. Interventions could also focus on reducing mothers’ mental burden and parenting stress (Luby et al., 2018) to free up their capacities to focus on their children's schooling. Being able to help children with their adaptation to school could, in turn, help mothers increase their own self‐efficacy and potentially improve their mental health.

Other intervention efforts could compensate for the elevated risk of cumulative parenting risk in the context of maternal depression symptoms by focusing on several different aspects of child functioning. The benefits of promoting multiple domains of child functioning at once is supported by research on resilience in the context of maternal depression, which showed that with each additional personal and social protective factor, adolescents showed more positive outcomes (Collishaw et al., 2016). Interventions broadly targeting positive youth development, such as social‐emotional learning approaches, have been shown to improve adaptive school behaviors, promote behavioral regulation, reduce maladaptive symptoms such as depression, and have short‐ and long‐term effects on academic success (Domitrovich et al., 2007; Riggs et al., 2006; Sorensen et al., 2016; Taylor et al., 2017). Also, our results suggest that parenting risk, early academic performance and child functioning are interrelated from early on. Supporting children and families during the transition to school is therefore key to foster children's optimal development.

### Strengths and limitations

We document longitudinal pathways from maternal depressive symptoms in childhood to adolescent academic performance in a sample representative of the surrounding community (i.e., a mid‐sized racially diverse city in the southeast of the US). Our results can therefore be generalized to mothers and their children from the community. Our study has several additional strengths, including its prospective longitudinal design spanning 13 years. We were able to model exposure to maternal depressive symptoms during the preschool period as a latent factor using measures of maternal depressive symptoms at three different time points (ages 2, 4, and 5) as indicators. This gave us an index of the average severity of maternal depressive symptoms over 3 years and provided a more accurate indicator of average exposure to depressive symptoms compared to a mean value. We also modeled the indirect effects of maternal depressive symptoms on adolescent academic performance in a structural equation modeling framework, which allowed us to estimate the significance not only of single paths but also of entire pathways. We used data from multiple informants to assess cumulative parenting risk, child functioning, and adolescent academic performance, which reduced same‐reporter bias. This is especially relevant as maternal depressive symptoms are known to influence maternal reports of child behavior (Goodman et al., 2011).

Our study also has limitations. First, even with our prospective longitudinal data, we could not establish causal associations. Second, our conceptualization of parenting risk as cumulative risk comprises aspects of parenting that are specific to children's schooling (e.g., parental involvement in children's education), and other more general adverse parenting behaviors (e.g., hostility). Although such a cumulative approach to parenting risk can capture the pervasive and heterogeneous associations that maternal depressive symptoms have with parenting, it may mask potential differences across parenting domains.

Third, our inclusion of cumulative parenting risk, child functioning, and adolescent academic performance as manifest variables in the path model does not consider potential measurement invariance for these constructs (Cole & Preacher, 2014). Fourth, we did not consider the role of fathers. While much of the research on parental depression focuses on maternal depression, more recent research has shown that fathers’ sensitivity can alleviate the effects of maternal depression on family functioning and child behavioral problems (Chang et al., 2007; Vakrat et al., 2018), and also that fathers’ depression can exacerbate the detrimental effects of maternal depression on child development (Landman‐Peeters et al., 2008).

Fifth, parenting behavior, the aspects of child functioning examined in this study, and academic performance are reciprocally interconnected across development (Claessens et al., 2015). For example, parental involvement in school is transactionally associated with children's academic achievement from preschool to adolescence (Sy et al., 2013). Also, higher levels of behavioral regulation in late childhood have been associated with subsequent improvements in social competence and mental health (King et al., 2013). Our supplementary analyses that included early academic performance also suggested bidirectional associations over time, which need to be investigated further in future research. Nevertheless, our findings suggest that maternal depressive symptoms in preschool are associated with pathways to poorer adolescent academic performance even when accounting for early academic performance.

Lastly, we considered parenting and child functioning as potential mechanisms in the pathways from maternal depression to adolescent academic performance. Other pathways such as more interpersonal difficulties with peers and teachers also seem plausible. A recent study that examined peer victimization as a potential mediator in the association between maternal depressive symptoms in early childhood and academic performance in pre‐adolescence could not confirm that, though (Ahun et al., 2020).

## CONCLUSION

Our findings suggest that maternal and child development are inextricably linked. We find that exposure to maternal depressive symptoms in childhood is indirectly linked to children's adolescent academic performance via cumulative parenting risk and child functioning. Although the effects are small, the findings highlight the importance of promoting and supporting mothers’ mental health and parenting to ensure optimal rearing conditions for child development. This is especially important when elevated symptoms of depression are associated with multiple parenting risks, which, in turn, have pervasive associations with child functioning and academic performance.

## ETHICS STATEMENT

The study was approved by the Institutional Review Board at The University of North Carolina at Greensboro (IRB protocol number 07‐0194).

## Supporting information

Supplementary Material
