# Supplementary material for: Pathways from maternal depressive symptoms to children’s academic performance in adolescence: A 13‐year prospective‐longitudinal study
Source: Child Dev. 2021 Oct 22;93(2):388–404. doi: 10.1111/cdev.13685 (PMC8930421; doi:10.1111/cdev.13685)
Supplement: Supplementary file 1 — Supplementary Material [file CDEV-93-388-s001.docx]

**Online Supplement**

**Pathways from Maternal Depressive Symptoms to Children’s Academic Performance in Adolescence: A 13-Year Prospective-Longitudinal Study**

**Supplement 1. Cumulative parenting risk without home chaos**

**
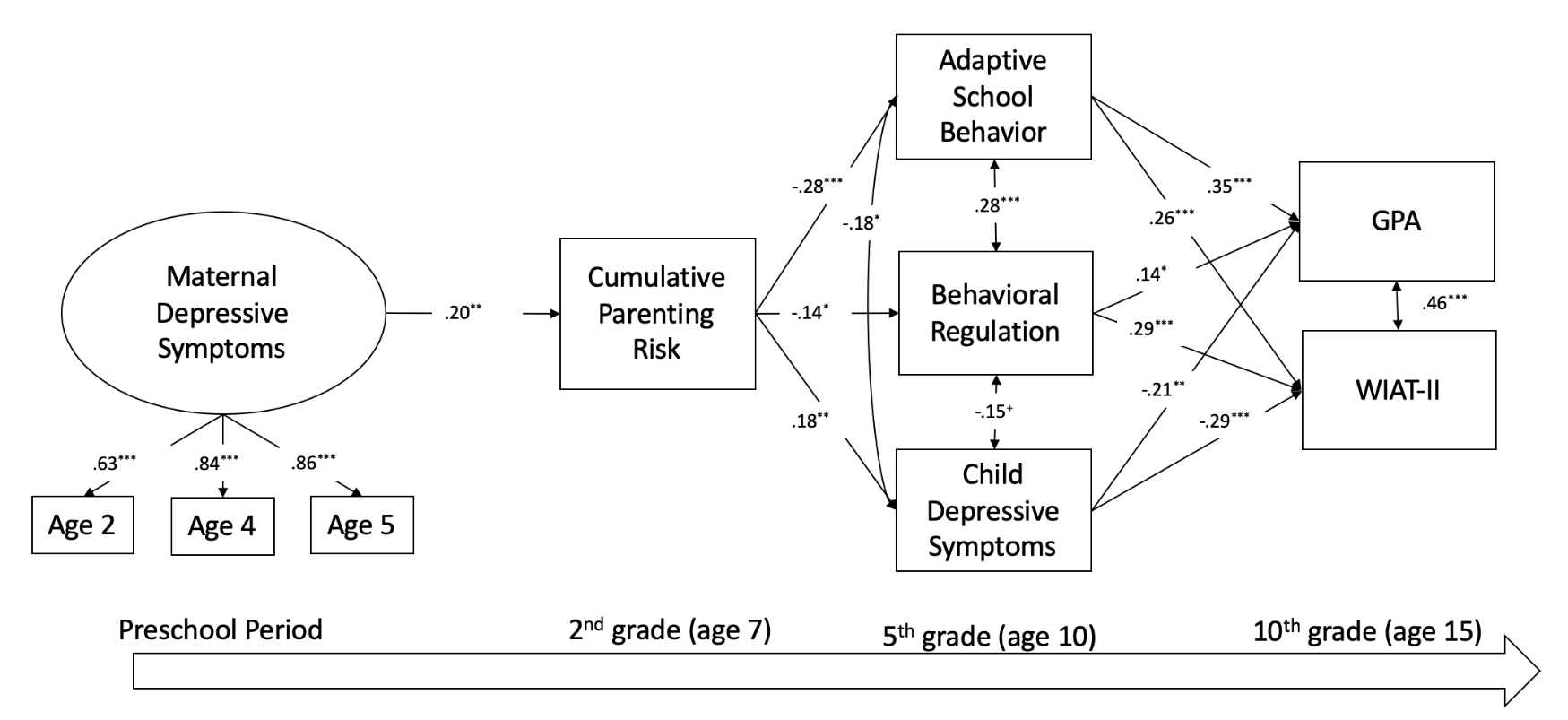
**

Figure S1. Standardized coefficients from a sensitivity analysis without home chaos as part of the cumulative parenting risk score.

*Note.* N = 389; χ^2^ (df) = 59.632 (50), CFI = .981, RMSEA = .030, SRMR = .044. Direct paths from maternal depressive symptoms to cumulative child functioning and academic performance outcomes, and from cumulative parenting risk to academic performance are not shown in the figure. The analysis is adjusted for SES, single motherhood, race (White vs. Other), sex, and child externalizing behaviors, all at age 2.

^+^ p < .10; ^*^ p < .05; ^**^ p < .01; *** p < .001.

**Supplement 2. Correlations between individual parenting behaviors**

Table S2

*Correlations between individual parenting behaviors*

| Variable | 1 | 2 | 3 | 4 | 5 | 6 |
| --- | --- | --- | --- | --- | --- | --- |
| 1) Hostility |  |  |  |  |  |  |
| 2) Low stimulation | **.31**** |  |  |  |  |  |
| 3) Chaos | -.01 | **.16*** |  |  |  |  |
| 4) Low involvement in school | .03 | **.25**** | -.02 |  |  |  |
| 5) Inconsistency | **.16**** | **.32**** | **.27**** | .10 |  |  |
| 6) Parenting Stress | **.17**** | **.25**** | **.49**** | .09 | **.32**** |  |
| 7) Cumulative parenting risk | **.42**** | **.62**** | **.51**** | **.32**** | **.55**** | **.54**** |

*Note.* Significant coefficients are bolded.

^*^ p < .05; ^**^ p < .01.

**Supplement 3. All coefficients from final model**

Table S3

*Standardized coefficients of the final model in the main manuscript (Figure 2)*

|  | β | SE | p |
| --- | --- | --- | --- |
| **Regressions** |  |  |  |
| MDS in preschool |  |  |  |
| SES (age 2) | **−.12** | .05 | .021 |
| Externalizing behavior (age 2) | **.42** | .06 | <.001 |
| Parenting risk (2^nd^ grade) |  |  |  |
| MDS in preschool | **.22** | .07 | .001 |
| SES (age 2) | **−.15** | .05 | .007 |
| Externalizing behavior (age 2) | ***.12*** | .06 | .054 |
| Single parenthood (age 2) | **.19** | .07 | .004 |
| ASB (5^th^ grade) |  |  |  |
| MDS in preschool | −.11 | .08 | .129 |
| Parenting risk (2^nd^ grade) | **−.24** | .06 | <.001 |
| Race (age 2) | **−.12** | .06 | .043 |
| Sex (age 2) | **.14** | .06 | .016 |
| Externalizing behavior (age 2) | **−.17** | .07 | .017 |
| BR (5^th^ grade) |  |  |  |
| MDS in preschool | **−*.13*** | .07 | .064 |
| Parenting risk (2^nd^ grade) | **−*.12*** | .06 | .067 |
| CDS (5^th^ grade) |  |  |  |
| MDS in preschool | .09 | .11 | .423 |
| Parenting risk (2^nd^ grade) | **.18** | .07 | .007 |
| Externalizing behavior (age 2) | .13 | .08 | .126 |
| GPA (10^th^ grade) |  |  |  |
| MDS in preschool | .06 | .07 | .366 |
| Parenting risk (2^nd^ grade) | −.08 | .06 | .204 |
| ASB (5^th^ grade) | **.35** | .07 | <.001 |
| BR (5^th^ grade) | **.14** | .06 | .006 |
| CDS (5^th^ grade) | **−.21** | .06 | .001 |
| Sex (age 2) | **.13** | .05 | .003 |
| Single parenthood (age 2) | **−.16** | .06 | .006 |
| WIAT-II (10^th^ grade) |  |  |  |
| MDS in preschool | .00 | .07 | .981 |
| Parenting risk (2^nd^ grade) | **−.16** | .06 | .004 |
| ASB (5^th^ grade) | **.26** | .05 | <.001 |
| BR (5^th^ grade) | **.29** | .05 | <.001 |
| CDS (5^th^ grade) | **−.29** | .06 | <.001 |
| SES (age 2) | ***.08*** | .05 | .080 |
| Race (age 2) | **−.18** | .05 | <.001 |
| **Covariances** |  |  |  |
| ASB and BR (5^th^ grade) | **.28** | .07 | <.001 |
| ASB and CDS (5^th^ grade) | **−.19** | .07 | .005 |
| BR and CDS (5^th^ grade) | **−.15** | .07 | .030 |
| GPA and WIAT-II (10^th^ grade) | **.45** | .06 | <.001 |

*Note.* Significant coefficients are bolded.

**Supplement 4. Accounting for the continuity of maternal depressive symptoms from childhood to adolescence**

A model that accounted for the continuity of maternal depressive symptoms from childhood to adolescence was specified. This model included all paths described in the data analysis section of the main manuscript. Furthermore, maternal depressive symptoms in 2^nd^ grade, 5^th^ grade, and 10^th^ grade were added to the model. Maternal depressive symptoms at one time point were included as predictors of maternal depressive symptoms at the following time point: maternal depressive symptoms in preschool were specified as a predictor of maternal depressive symptoms in 2^nd^ grade, which were specified as a predictor of maternal depressive symptoms in 5^th^ grade, which were specified as a predictor of maternal depressive symptoms in 10^th^ grade. Maternal depressive symptoms in 2^nd^ grade were specified as a predictor of children’s functioning in 5^th^ grade, and children’s academic performance in 10^th^ grade. Maternal depressive symptoms in 5^th^ grade were specified as a predictor of 10^th^ grade academic performance. Maternal depressive symptoms at one point in time were allowed to covary with constructs assessed at the same point in time. The conceptual figure of the model just described can be seen in Figure S4. At first, all covariates were included as predictors of all variables in the model. Non-significant covariates were then removed, until only covariates with p<.10 were included in the final model.


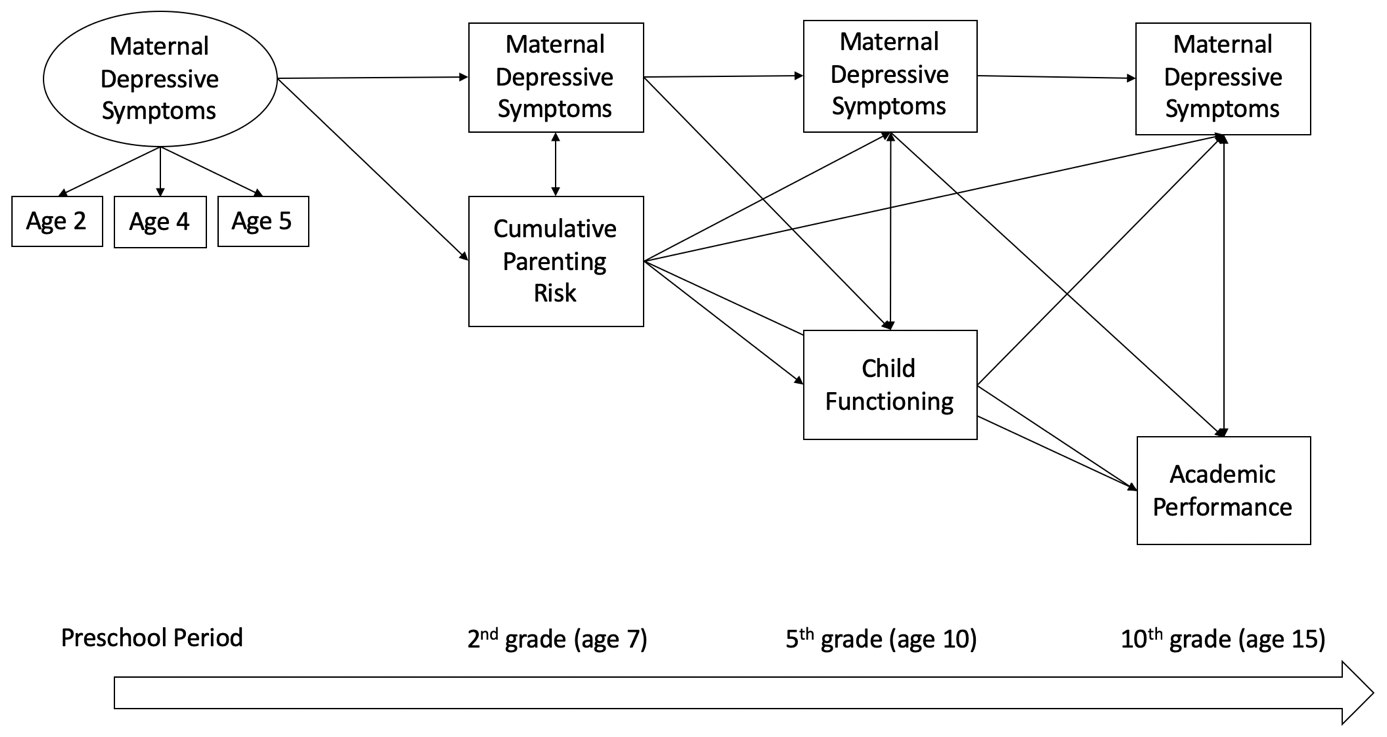


**Figure S4.** Conceptual model of the post-hoc analysis that accounted for the continuity of maternal depressive symptoms

Maternal depressive symptoms were moderately stable across time (see Table S4.1 for model coefficients). Cumulative parenting risk in 2^nd^ grade was uniquely associated with child functioning in 5^th^ grade, after accounting for the influence of maternal depressive symptoms in 2^nd^ grade. In fact, maternal depressive symptoms in 2^nd^ grade were not significantly associated with child functioning in 5^th^ grade. All three indicators of child functioning in 5^th^ grade were significantly associated with academic performance in 10^th^ grade independent of maternal depressive symptoms in 5^th^ grade. Maternal depressive symptoms in 5^th^ grade were not predictive of 10^th^ grade academic achievement. There are significant total indirect effects from maternal depressive symptoms in preschool to 10^th^ grade GPA, −0.051, 95% CI = (−0.089, −0.022), and standardized achievement, −0.028, 95% CI= (−0.053, −0.004). All unique indirect effects that were identified in the main model remained significant (see Table S4.2). These findings strengthen our conclusion that maternal depressive symptoms in the preschool period are associated with pathways to poorer academic performance in 10^th^ grade through cumulative parenting risk and child functioning.

**Table S4.1**

*Standardized coefficients of the model adjusting for the continuity of maternal depressive symptoms*

|  | β | SE | p-value |
| --- | --- | --- | --- |
| **Regressions** |  |  |  |
| MDS in preschool |  |  |  |
| SES (age 2) | **-0.12** | 0.05 | 0.027 |
| Externalizing behavior (age 2) | **0.41** | 0.06 | 0.000 |
| Parenting risk (2^nd^ grade) |  |  |  |
| MDS in preschool | **0.23** | 0.07 | 0.001 |
| SES (age 2) | **-0.15** | 0.05 | 0.006 |
| Externalizing behavior (age 2) | **0.12** | 0.06 | 0.042 |
| Single parenthood (age 2) | **0.18** | 0.07 | 0.006 |
| MDS in 2^nd^ grade |  |  |  |
| MDS in preschool | **0.76** | 0.05 | 0.000 |
| ASB (5^th^ grade) |  |  |  |
| MDS in preschool | -0.09 | 0.13 | 0.509 |
| MDS in 2^nd^ grade | -0.05 | 0.12 | 0.696 |
| Parenting risk (2^nd^ grade) | **-0.24** | 0.06 | 0.000 |
| Race (age 2) | **-0.12** | 0.06 | 0.040 |
| Sex (age 2) | **0.13** | 0.06 | 0.020 |
| Externalizing behavior (age 2) | **-0.16** | 0.07 | 0.027 |
| BR (5^th^ grade) |  |  |  |
| MDS in preschool | -0.14 | 0.12 | 0.250 |
| MDS in 2^nd^ grade | 0.00 | 0.12 | 0.987 |
| Parenting risk (2^nd^ grade) | ***-0.11*** | 0.07 | 0.083 |
| CDS (5^th^ grade) |  |  |  |
| MDS in preschool | 0.07 | 0.15 | 0.624 |
| MDS in 2^nd^ grade | 0.16 | 0.14 | 0.241 |
| Parenting risk (2^nd^ grade) | **0.17** | 0.07 | 0.015 |
| MDS in 5^th^ grade |  |  |  |
| MDS in 2^nd^ grade | **0.42** | 0.08 | 0.000 |
| Parenting risk (2^nd^ grade) | **0.14** | 0.07 | 0.043 |
| GPA (10^th^ grade) |  |  |  |
| MDS in preschool | ***0.22*** | 0.11 | 0.049 |
| MDS in 2^nd^ grade | -0.13 | 0.09 | 0.155 |
| MDS in 5^th^ grade | -0.08 | 0.08 | 0.308 |
| Parenting risk (2^nd^ grade) | -0.04 | 0.07 | 0.506 |
| ASB (5^th^ grade) | **0.35** | 0.07 | 0.000 |
| BR (5^th^ grade) | **0.14** | 0.06 | 0.018 |
| CDS (5^th^ grade) | **-0.19** | 0.07 | 0.004 |
| SES (age 2) | **0.13** | 0.06 | 0.029 |
| Sex (age 2) | **0.14** | 0.05 | 0.003 |
| Single parenthood (age 2) | **-0.14** | 0.06 | 0.018 |
| WIAT-II (10^th^ grade) |  |  |  |
| MDS in preschool | -0.00 | 0.11 | 0.971 |
| MDS in 2^nd^ grade | 0.01 | 0.08 | 0.941 |
| MDS in 5^th^ grade | 0.00 | 0.06 | 0.942 |
| Parenting risk (2^nd^ grade) | **-0.16** | 0.06 | 0.009 |
| ASB (5^th^ grade) | **0.25** | 0.05 | 0.000 |
| BR (5^th^ grade) | **0.29** | 0.05 | 0.000 |
| CDS (5^th^ grade) | **-0.27** | 0.07 | 0.000 |
| SES (age 2) | **0.13** | 0.05 | 0.011 |
| Race (age 2) | **-0.19** | 0.05 | 0.000 |
| MDS (5^th^ grade) |  |  |  |
| ASB (5^th^ grade) | 0.05 | 0.06 | 0.443 |
| BR (5^th^ grade) | -0.09 | 0.06 | 0.137 |
| CDS (5^th^ grade) | 0.08 | 0.09 | 0.347 |
| MDS (5^th^ grade) | **0.50** | 0.07 | 0.000 |
| Externalizing Symptoms (age 2) | **0.17** | 0.06 | 0.002 |
| **Covariances** |  |  |  |
| CR and MDS in 2^nd^ grade | 0.13 | 0.09 | 0.161 |
| ASB and BR (5^th^ grade) | **0.29** | 0.07 | 0.000 |
| ASB and CDS (5^th^ grade) | **-0.19** | 0.07 | 0.005 |
| BR and CDS (5^th^ grade) | **-0.16** | 0.07 | 0.025 |
| ASB and MDS in 5^th^ grade | -0.05 | 0.07 | 0.495 |
| BR and MDS in 5^th^ grade | -0.01 | 0.07 | 0.854 |
| Dep and MDS in 5^th^ grade | 0.04 | 0.11 | 0.753 |
| GPA and WIAT-II (10^th^ grade) | **0.46** | 0.06 | 0.000 |
| GPA and MDS in 10^th^ grade | -0.01 | 0.10 | 0.948 |
| WIAT and MDS in 10^th^ grade | 0.10 | 0.10 | 0.313 |

*Note.* Significant coefficients are bolded. MDS = maternal depressive symptoms, ASB = adaptive school behaviors, BR = behavioral regulation, CDS = child depressive symptoms, GPA = grade point average, WIAT = standardized achievement. χ^2^ (df) = 158.79 (68), RMSEA = 0.059, CFI = 0.927, SRMR = 0.059.

**Table S4.2**

*Unstandardized Estimates of Significant Indirect Effects, Standard Errors, and 95% Bias-Corrected Bootstrap Confidence Intervals (5,000 draws)*

|  |  |  | Confidence Interval | |
| --- | --- | --- | --- | --- |
| Indirect paths | Estimate | SE | Lower | Upper |
| MDS 🡪PR🡪ASB🡪GPA | -0.004 | 0.002 | -0.008 | -0.001 |
| MDS🡪PR🡪ASB🡪WIAT | -0.002 | 0.001 | -0.005 | -0.001 |
| MDS 🡪CR🡪ASB | -0.458 | 0.174 | -0.882 | -0.189 |
| MDS 🡪PR🡪CDS | 0.009 | 0.004 | 0.002 | 0.019 |
| MDS🡪PR🡪WIAT | -0.005 | 0.002 | -0.011 | -0.001 |

*Note.* MDS = maternal depressive symptoms, PR = Parenting Risk, ASB = adaptive school behaviors, CDS = child depressive symptoms, GPA = grade point average, WIAT = standardized achievement.

**Supplement 5. Accounting for the continuity of academic performance in 2^nd^ grade**

A model that accounted for the continuity of academic performance from childhood to adolescence was specified. This model included all paths described in the data analysis section of the main manuscript. Participants’ standardized achievement in 2^nd^ grade was included as an indicator of early academic performance. A path from maternal depressive symptoms in the preschool period to 2^nd^ grade academic performance was modelled. Academic performance in 2^nd^ grade was further specified as a predictor of child functioning in 5^th^ grade and academic performance in 10^th^ grade. The conceptual figure of the model can be seen in Figure S5. At first, all covariates were included as predictors of all variables in the model. Non-significant covariates were then removed, until only covariates with p<.10 were included in the final model.


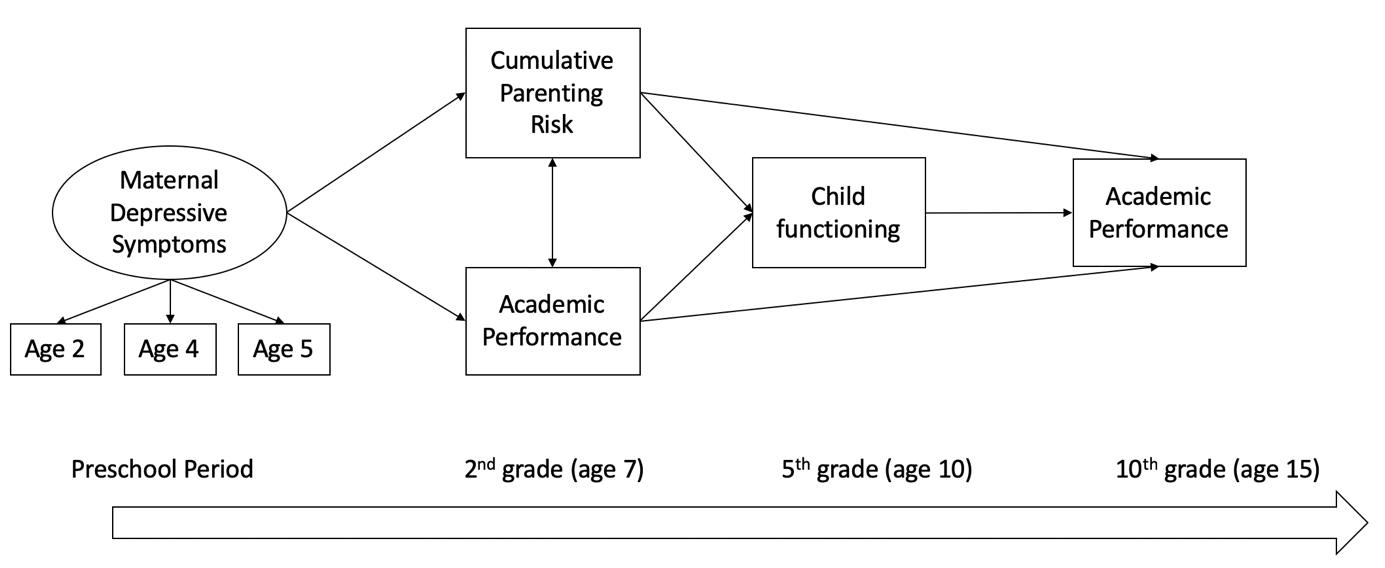


**Figure S5.** Conceptual model of the post-hoc analysis including the continuity of academic performance in 2^nd^ grade.

Maternal depressive symptoms in the preschool period were not associated with standardized achievement in 2^nd^ grade (see Table S5.1 for all model coefficients). Cumulative parenting risk was only uniquely associated with children’s adaptive school behavior once early academic performance was taken into account, and no longer with children’s behavioral regulation and depressive symptoms. Academic performance in 2^nd^ grade was significantly associated with all aspects of child functioning. All aspects of child functioning remained predictive of standardized achievement in 10^th^ grade when standardized achievement in 2^nd^ grade was included in the model, but only adaptive school behavior remained predictive of 10^th^ grade GPA. The total indirect effects from maternal depressive symptoms in preschool to 10^th^ grade GPA, −0.021, 95% CI= (−0.040, −0.005), and standardized achievement, −0.019, 95% CI = (−0.011, −0.001), remained significant. These findings suggest that some of the associations of cumulative parenting risk and child functioning can be accounted for by early academic performance and highlight the dynamic interconnectedness of parenting risk, child functioning and academic performance from the early school years on and into adolescence. Our main takeaways, namely, that maternal depressive symptoms in preschool are associated with pathways to adolescent academic performance, remain unchanged. Specifically, children’s adaptive school behaviors remain especially important in the pathway from maternal depressive symptoms to adolescent academic performance (see Table S5.2 for significant indirect effects).

**Table S5.1**

*Standardized coefficients of the model adjusting for the continuity of academic performance*

|  | β | SE | p-value |
| --- | --- | --- | --- |
| **Regressions** |  |  |  |
| MDS in preschool |  |  |  |
| SES (age 2) | **-0.12** | 0.05 | 0.025 |
| Externalizing behavior (age 2) | **0.43** | 0.06 | 0.000 |
| Parenting risk (2^nd^ grade) |  |  |  |
| MDS in preschool | **0.23** | 0.07 | 0.001 |
| SES (age 2) | **-0.14** | 0.05 | 0.008 |
| Externalizing behavior (age 2) | ***0.12*** | 0.06 | 0.064 |
| Single parenthood (age 2) | **0.19** | 0.07 | 0.004 |
| Academic performance in 2^nd^ grade |  |  |  |
| MDS in preschool | -0.06 | 0.08 | 0.496 |
| SES (age 2) | **0.19** | 0.06 | 0.002 |
| Externalizing behavior (age 2) | **-0.26** | 0.07 | 0.000 |
| Single parenthood (age 2) | **-0.15** | 0.07 | 0.038 |
| ASB (5^th^ grade) |  |  |  |
| MDS in preschool | ***-0.13*** | 0.07 | 0.066 |
| Parenting risk (2^nd^ grade) | **-0.18** | 0.07 | 0.008 |
| Academic performance in 2^nd^ grade | **0.38** | 0.06 | 0.000 |
| Sex (age 2) | **0.12** | 0.06 | 0.034 |
| BR (5^th^ grade) |  |  |  |
| MDS in preschool | -0.04 | 0.08 | 0.579 |
| Parenting risk (2^nd^ grade) | -0.02 | 0.06 | 0.761 |
| Academic performance in 2^nd^ grade | **0.43** | 0.07 | 0.000 |
| Sex (age 2) | ***-0.10*** | 0.06 | 0.084 |
| CDS (5^th^ grade) |  |  |  |
| MDS in preschool | 0.08 | 0.09 | 0.375 |
| Parenting risk (2^nd^ grade) | 0.10 | 0.07 | 0.138 |
| Academic performance in 2^nd^ grade | **-0.40** | 0.06 | 0.000 |
| GPA (10^th^ grade) |  |  |  |
| MDS in preschool | 0.07 | 0.07 | 0.302 |
| Parenting risk (2^nd^ grade) | -0.06 | 0.06 | 0.300 |
| Academic performance in 2^nd^ grade | **0.24** | 0.09 | 0.008 |
| ASB (5^th^ grade) | **0.30** | 0.07 | 0.000 |
| BR (5^th^ grade) | 0.07 | 0.07 | 0.271 |
| CDS (5^th^ grade) | ***-0.14*** | 0.07 | 0.060 |
| Sex (age 2) | ***0.10*** | 0.05 | 0.051 |
| Single parenthood (age 2) | **-0.14** | 0.06 | 0.015 |
| WIAT-II (10^th^ grade) |  |  |  |
| MDS in preschool | 0.02 | 0.07 | 0.809 |
| Parenting risk (2^nd^ grade) | **-0.15** | 0.06 | 0.008 |
| Academic performance in 2^nd^ grade | **0.43** | 0.08 | 0.000 |
| ASB (5^th^ grade) | **0.19** | 0.06 | 0.001 |
| BR (5^th^ grade) | **0.16** | 0.05 | 0.001 |
| CDS (5^th^ grade) | **-0.14** | 0.06 | 0.036 |
| SES (age 2) | **-0.10** | 0.04 | 0.024 |
| Race (age 2) | **-0.18** | 0.04 | 0.000 |
| **Covariances** |  |  |  |
| ASB and BR (5^th^ grade) | **0.17** | 0.07 | 0.020 |
| ASB and CDS (5^th^ grade) | -0.07 | 0.07 | 0.313 |
| BR and CDS (5^th^ grade) | 0.00 | 0.08 | 0.997 |
| Parenting risk and academic performance in 2^nd^ grade | -0.11 | 0.07 | 0.119 |
| GPA and WIAT-II (10^th^ grade) | **0.40** | 0.07 | 0.000 |

*Note.* χ^2^ (df)=54.895(50), RMSEA=0.016, CFI=0.995, SRMR=0.039.

**Table S5.2**

*Unstandardized Estimates of Significant Indirect Effects, Standard Errors, and 95% Bias-Corrected Bootstrap Confidence Intervals (5,000 draws)*

|  |  |  | Confidence Interval | |
| --- | --- | --- | --- | --- |
| Indirect paths | Estimate | SE | Lower | Upper |
| MDS 🡪 PR 🡪 ASB 🡪GPA | -0.002 | 0.001 | -0.006 | -0.001 |
| MDS 🡪 PR 🡪 WIAT | -0.005 | 0.002 | -0.011 | -0.001 |
| MDS 🡪 PR 🡪 ASB | -0.354 | 0.163 | -0.764 | -0.098 |

*Note.* MDS = maternal depressive symptoms, PR = Parenting Risk, ASB = adaptive school behaviors, GPA = grade point average, WIAT = standardized achievement.
